# The Boston Dental College

**Published:** 1869-06

**Authors:** 


### THE BOSTON DENTAL COLLEGE.

The following we take from the *Boston Daily Advertiser* and publish, that our readers may know the true state of the matter. Rumor will usually very much color such things. It is a matter of regret that there was an occasion for any such proceedings, and yet it is gratifying to know that there is a disposition in the minds of some to correct irregularities.

Perhaps there has been, in nearly all existing Dental Colleges, too much laxity in the matter here complained of. We make no charges in this respect. We feel confident that the wants of the profession, in an educational aspect, requires a higher standard than at present exists .

SUPREME JUDICIAL COURT—Suffolk, ss.—June 19.

Colt, J.—*The Attorney General by Relation vs. The Boston Dental College et al.*—This was an information at the relation of John P. Ordway and others, constituting a minority of the Board of Trustees of the Boston Dental College, to restrain the respondents, the majority of the Board, from conferring the degree of "Doctor of Dental Surgery" upon certain candidates who have been recommended for such degree by the faculty of the College. By the act of incorporation, passed June 3, 1868, the Trustees of the College have authority to confer the degree of "Doctor of Dental Surgery" upon candidates therefor who, upon satisfactory examination by the faculty, have been recommended to the trustees for the degree, provided the candidates shall have devoted three years to the study of Dentistry with a practitioner of Dental Surgery, who shall be approved by the faculty, or shall have been in the practice of Dental Surgery for eight years, *including two full courses of lectures*, the last of which to be pursued in the Boston Dental College.

At the hearing on Saturday, on a motion for an injunction, it appeared that the college was opened for the instruction of students, in the month of September last, and that it has been in

